# Neural substrates of anticipatory motor adaptation for object lifting

**DOI:** 10.1038/s41598-020-67453-0

**Published:** 2020-06-26

**Authors:** Michelle Marneweck, Scott T. Grafton

**Affiliations:** 1 Monash University, Clayton, VIC Australia; 2 Department of Psychological & Brain Sciences, University of California, Santa Barbara, Santa Barbara, CA 93106 USA

**Keywords:** Neurophysiology, Motor control

## Abstract

Anticipatory force control is a fundamental means by which humans stave off slipping, spilling, and tilting disasters while manipulating objects. This control must often be adapted due to changes in an object’s dynamics (e.g. a lighter than expected mug of coffee) or its relation with involved effectors or digits (e.g. lift a mug with three vs. five digits). The neural processes guiding such anticipatory and adaptive control is understudied but presumably operates along multiple time scales, analogous to what has been identified with adaptation in other motor tasks, such as perturbations during reaching. Learning of anticipatory forces must be ultrafast to minimize tilting a visually symmetric object towards its concealed asymmetric center of mass (CoM), but slower when the CoM is explicitly and systematically switched from side to side. Studying the neural substrates of this latter slower learning process with rapid multiband brain imaging, in-scanner kinematics and Bayesian pattern component modelling, we show that CoM-specific pattern distances increase with repeated CoM switching exposures and improved learning. The cerebellum showed the most prominent effects, fitting with the idea that it forms a stored internal model that is used to build and update anticipatory control. CoM-specific pattern distances were present 24 h later, in line with the presence of consolidation effects.

## Introduction

Numerous behavioral and computational studies demonstrate that a wide variety of motor behaviors can be adapted to a constantly fluctuating environment and that this learning occurs on multiple time scales^1^. For example, prism adaptation, force field adaptation and visuomotor rotation all can be described by a combination of fast and slow learning components. The same general idea of multiple time scales is likely to be operating when people manipulate objects. In this case, when an object’s dynamics are mismatched with expectations, the lift forces of the digits adapt extremely rapidly to minimize roll or other aspects of movement error^2^. For example, it takes only three trials to successfully lift a visually symmetrical inverted T-shaped object without tilting it towards its concealed asymmetric center of mass (CoM). This is accomplished by an anticipatory partitioning of digit lift forces at lift onset (i.e. more force by the digit closest to the CoM), which generates an appropriate torque counteracting the offset CoM^3^. This improvement over just a few trials can be described as an ultrafast form of adaptation^4,5^. When the object CoM is then switched from one side to the other (which alters the relation between the involved digits and the object), the movement errors re-emerge, suggesting that ultrafast adaptation is not immediately generalizable to different CoM offsets and appropriate lift forces must be re-acquired^6,7^. Critically, this failure to generalize to a new CoM occurs despite a subject's explicit perceptual awareness of the switch (seeing the object being rotated) and an intact knowledge of the CoM (correctly pointing to it)^7,8^. On the other hand, if the subject continues to experience alternations of a CoM, there is evidence for learning taking place on a second slower time scale. Over a repeated exposure where the object CoM is systematically switched, the movement errors on the first switch trial diminish, suggesting it is possible to eventually learn something about the interaction of lift forces and object dynamics^3^. In this study, we are interested in the learning occurring on this relatively slower time scale. The neuroanatomic substrates underlying object manipulation and learning on this time scale are not known, which we examine here using kinematics, brain imaging and pattern component modeling.

Our experimental approach begins by extracting cortical and subcortical activity obtained by functional imaging from brain regions that distinguish differences in successfully manipulating objects with different CoMs^9,10^. After a small block of trials of lifting an object with one CoM, a given region will demonstrate a multivoxel spatial pattern that is separable from the pattern that is present after the same number of trials with the alternate CoM. The within block changes of brain activity reflect ultrafast adaptation, and the CoM-specific patterns are described elsewhere^10^. Here, we consider the slower adaptation as each of the CoMs is learned over repeated blocks of trials. To map this learning-related change, we focus on the pattern distance in a brain region at the moment the CoM is reversed. At the switch, the brain needs to adopt the alternate pattern. In this study, we test whether the ability to switch to the new appropriate pattern improves with repeated exposures. If this were true, then the pattern distance between the pre- and post-switch activity (reflecting the two CoMs) should increase over blocks of trials with multiple reversals. We predicted these differences between earlier and later blocks to be particularly apparent in cerebellum, given its role in storing internal models that are used to build and update predictive control, e.g.^11,12^. In other words, CoM-specific patterns would be less discriminable during early sensorimotor learning, when the internal model of one CoM might interfere or be incorrectly implemented after the CoM switch. Critically, by using a rapid multiband imaging approach, we can test for these effects before lift onset, and thus, examine anticipatory-related activity independent of motor execution and feedback processes. We repeat these analyses on a subset of subjects returning 24 h later and test for behavioral retention, a measure of consolidation. Depending on whether learning is consolidated 24 h later, we then have the opportunity to test if this is reflected in the corresponding CoM-specific fMRI pattern distances.

## Results

**Figure 1:**
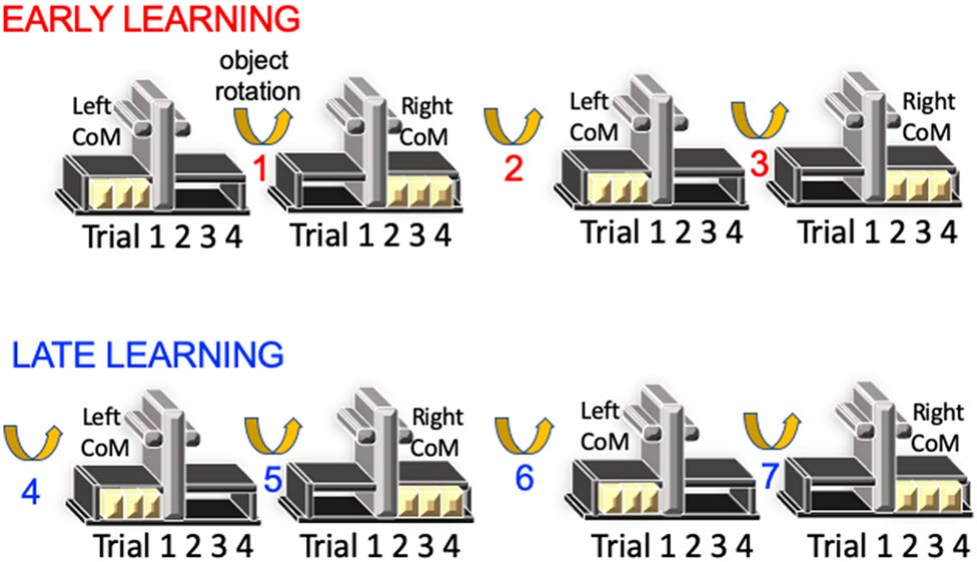
An illustration of the object and study design. Subjects lay supine in the scanner as they reached grasped and lifted an object with an asymmetric center of mass (CoM) that switched seven times every four trials from one side to the other.

Following a T1-structural scan, BOLD contrast was measured while 16 subjects in a supine position learned to reach, grasp, and lift a novel object with an asymmetric CoM with the goal of minimizing its tilt or roll (see Fig. 1). The experimenter rotated the object every 4 trials such that the CoM switched between the left and the right. The extent to which learning was transferred or generalized between CoMs was assessed behaviorally via in-scanner kinematic measurements of object roll. Following preprocessing of structural and fMRI data, first-level deconvolution-based general linear models (GLMs) captured 800-ms time bins of cortical and subcortical activity for a total of 7.2 s starting 800 ms before lift onset for each pre- and post-switch trial at each of the 7 switching blocks. Given the average 4–6 s delay of the hemodynamic response, this method predominantly tracked activity relating to pre-lift anticipatory behavior, as was done previously^9,10^. GLM-derived beta weights were then subsequently used in a Bayesian implementation of representational similarity analyses, i.e., variational representational similarity analyses (vRSA), to assess the extent to which multivoxel spatial patterns in prespecified regions of interest (ROIs) were more sensitive to differences between pre- and the first post-switch trials in later than earlier blocks of sensorimotor learning. This procedure and the analyses were repeated in a subset of 10 subjects who returned 24 h later.

### Behavioral errors are greater after CoM switches in early than later blocks in day 1 but not day 2

**Figure 2:**
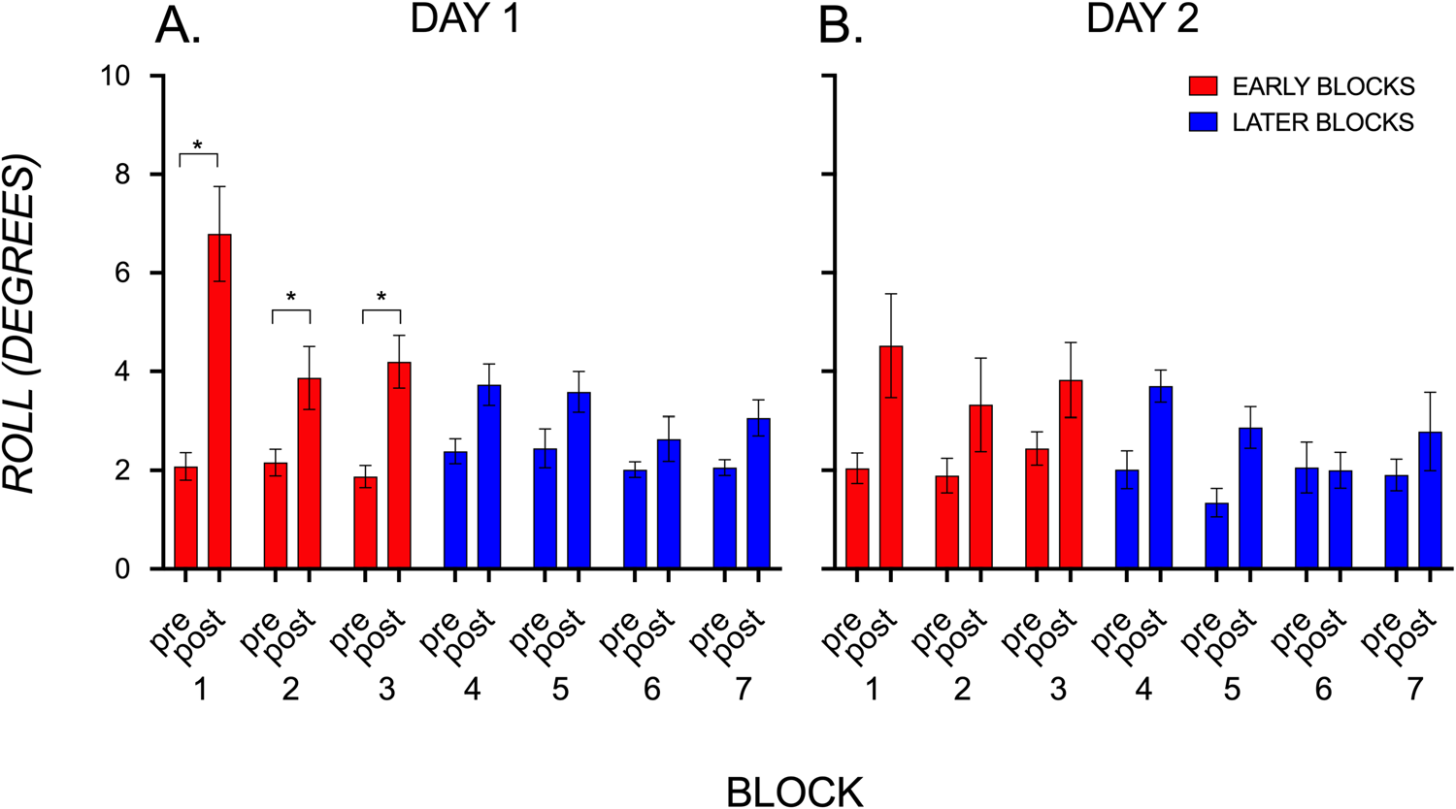
Kinematic tracking of sensorimotor learning. Roll (in degrees) on trials before and after a switch in the center of mass results in (**A**) greater errors during early blocks (1 to 3) and success in later blocks (4 to 7) on day 1, and (**B**) similar errors across blocks on day 2. Consistent with a consolidation effect, error is smaller on the first block’s post-switch trial on day 2 than on day 1.

Figure 2A shows kinematic roll data for seven sets of pre- and post-switch trials. There was a statistically significant main effect of CoM switch (*F*(1,15) = 50.80, *p* < 0.0001) and an effect of block (*F*(3.50, 52.46) = 4.23, *p* = 0.007). However, this block effect did not survive multiple comparison corrections. A significant interaction (*F*(3.08, 46.18) = 5.85, *p* = 0.002) showed that the pre- and post-switch trials were significantly different in blocks 1 to 3, after adjusting for multiple comparisons. This shows that by the block 4 (trial 13), subjects learned to counter the torque in the direction of the external torque, with little interference from the preceding block of trials.

Figure 2B shows kinematic roll data for seven sets of pre- and post-switch trials for 10 subjects who returned to the lab 24 h later to complete the same object lifting task during an fMRI scan. Most striking were substantially lower errors on the first post-switch trial within the first block on day 2 compared to the same trial in the first block of day 1. When we repeated the behavioral analyses of day 1, we found no more interaction or block effects, and a smaller effect of switch (*F*(1,9) = 15.26, *p* = 0.004). Notably, the significant difference between pre- and post-switch trials of block 1 on day 1 (*p* = 0.0018) was not present on day 2 (*p* = 0.27). Thus, while errors were still somewhat elevated across all post-switch trials, the effect was substantially smaller as a result of consolidation or partial retention from day 1 learning.

We next analyzed multivoxel spatial patterns in pre- and post-switch trials in early and later blocks of day 1 to test for evidence that an ROI is differentially sensitive to CoM in early compared to later blocks. In addition, given our behavioral evidence for partial consolidation or retention of learning 24 h later, we also tested whether there was a corresponding effect on CoM-specific pattern distances on day 2. That is, would CoM-specific patterns already be evidently present in early blocks of trials on day 2 and to a similar extent as that seen on later blocks from day 2?

### CoM-specific patterns are more discriminable during late than early attempts of lifting objects of different CoMs on day 1 but not on day 2

We used vRSA to assess the evidence for condition-specific patterns of responses distributed over voxels in a given ROI^13^. This method is an extension of the more traditional RSA approach for comparing between-condition differences in multivoxel spatial patterns in a given ROI. It is particularly useful for simultaneously assessing patterns of distances across conditions in a representational matrix. It allows us to test if the posterior probability of the underlying pattern distances is consistent with linear contrasts of the conditions, a type of pattern component modeling. The approach takes into account all proposed contrasts and their interactions. The vRSA contrasts described below were run on data collected on day 1 and on a subset of subjects who repeated the testing procedure 24 h later.

**Figure 3:**
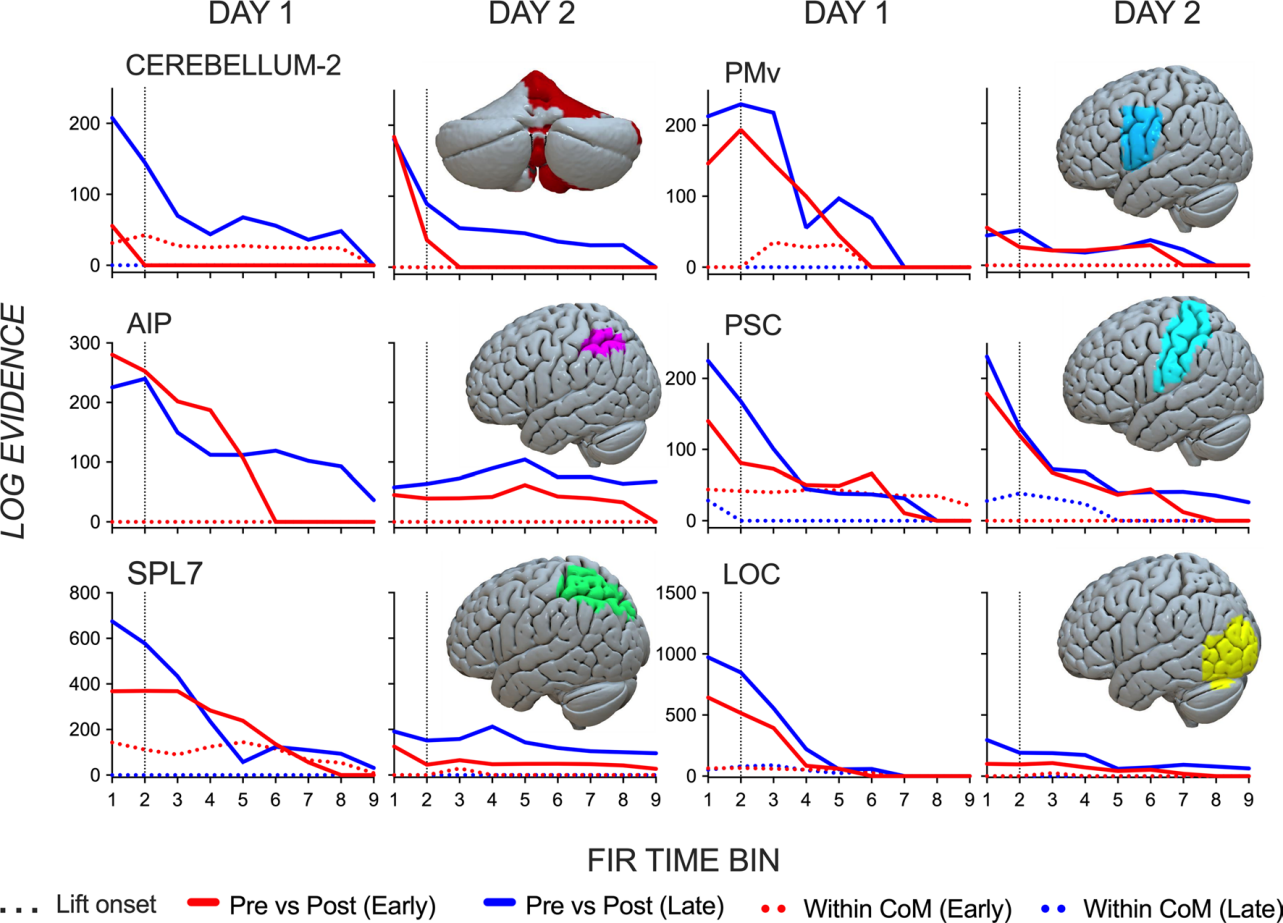
Model evidence for learning effects based on each region’s response pattern. Log evidence values reflecting the evidence for contrasts between pre- and post-switch trials (solid lines) of manipulating objects with a switching center of mass (CoM), and between blocks of trials with the same CoM (dotted lines) during early (block 1 to 3; red) and late learning (block 4 to 7; blue) on day 1 and day 2 in regions of interest (ROIs) for each of the 9 finite impulse response (FIR) time bins. Lift onset aligned with the start of FIR bin 2, and is depicted by a black vertical dotted line. ROIs are displayed on the MNI-152 atlas using the visualization software, Surf Ice (https://www.nitrc.org/projects/surfice/). On day 1, CoM-specific pattern differentiation increases in later than early blocks in early FIR bins, and in particular in cerebellum. These CoM-specific patterns remain present in the early block of day 2, consistent with a behavioral consolidation effect.

The outcome of each specified contrast is a log evidence (Bayes factor) value, which quantifies the evidence for a given contrast to contribute to dissociable activity patterns in a given ROI. Figure 3 shows log evidence values that quantify the evidence for four model contrasts to contribute to dissociable activity patterns across 9 successive time bins (starting before lift onset) in a set of predefined ROIs. A relative log evidence value (i.e. Bayes factor) of 3 and above matches a “substantial” amount of evidence that a contrast of interest contributes to a region’s response pattern^14,15^. Two of the four model comparisons tested our hypothesis that voxel spatial pattern differences for pre- and post-switch trials (reflecting two CoM conditions) would increase with learning by contrasting conditions in which the object was lifted before and after the CoM switch during early and later blocks (when learning was absent and present, respectively). Critically, we were interested in the *change* in spatial voxel patterns between CoM conditions from early to later blocks, rather than the magnitude of the difference in spatial voxel patterns between CoM conditions during the early or late block. If these patterns became more different from each other with learning, it would suggest that they represent that which is unique or specific to a given condition (CoM) when the task is learned. Two additional model comparisons contrasted within CoM pattern differences during early and later blocks, thereby testing a region’s ‘baseline’ sensitivity to differences between these time points, irrespective of performance or learning improvements. We focus on early than later time bins (i.e. deconvolution-based fMRI data in early finite impulse response time bins). Early time bins more definitively demarcate anticipatory feedforward from feedback processes before the hemodynamic responses relating to lift onset peaks from 4 s (bin 7 onwards).

On day 1, there is a marked difference in the degree to which CoM patterns are different from each other in early and later blocks. In the early block, and in early time bins in particular, the evidence for CoM-specialized patterns is lower than that seen in later blocks in most ROIs. In the later block, the sensitivity to the pre- and post-switch effect increases substantially and particularly in early time bins compared to that seen in the early block. This early vs. late effect is most prominent in cerebellum, where a CoM-specific difference is noticeably absent in the early block. As has been shown previously, this suggests these regions contain CoM-specific pattern representations beginning prior to lift onset that successfully minimize object tilting with varying CoMs^9,10^.

On day 2, the magnitude of Bayes log values indicating the evidence for CoM conditions to contribute to dissociable activity patterns are more similar between early and later blocks than that seen in the day 1 data. That is, there is evidence that all regions display an appropriate CoM-specific pattern at the start of the session. As previously stated, this neural specificity is similarly reflected in the behavioral results indicative of consolidation or retention where the error after the first CoM switch is substantially diminished than that seen in day 1.

Figure 3 also shows a pair of baseline contrasts that assessed whether neural representations of manipulating the same CoM, according to the same success, would vary between early and late sensorimotor learning (i.e. pattern differentiation differences between early and late blocks, irrespective of learning). Unlike the between-CoM contrasts, there was much lower evidence for within CoM pattern differences across all time bins during both early and later blocks on both days. Interestingly, there was a slight increase in pattern distances in early than later blocks in some ROIs in day 1, which dissipated 24 h later. These within-CoM pattern differences in the early block of day 1 were evidently minimal compared to our main contrasts of interest, and completely diminished by day 2, which rules out that an evolution of CoM-specific patterns over time reflect a time-modulated factor that is unrelated to learning CoM-specific force control.

**Figure 4:**
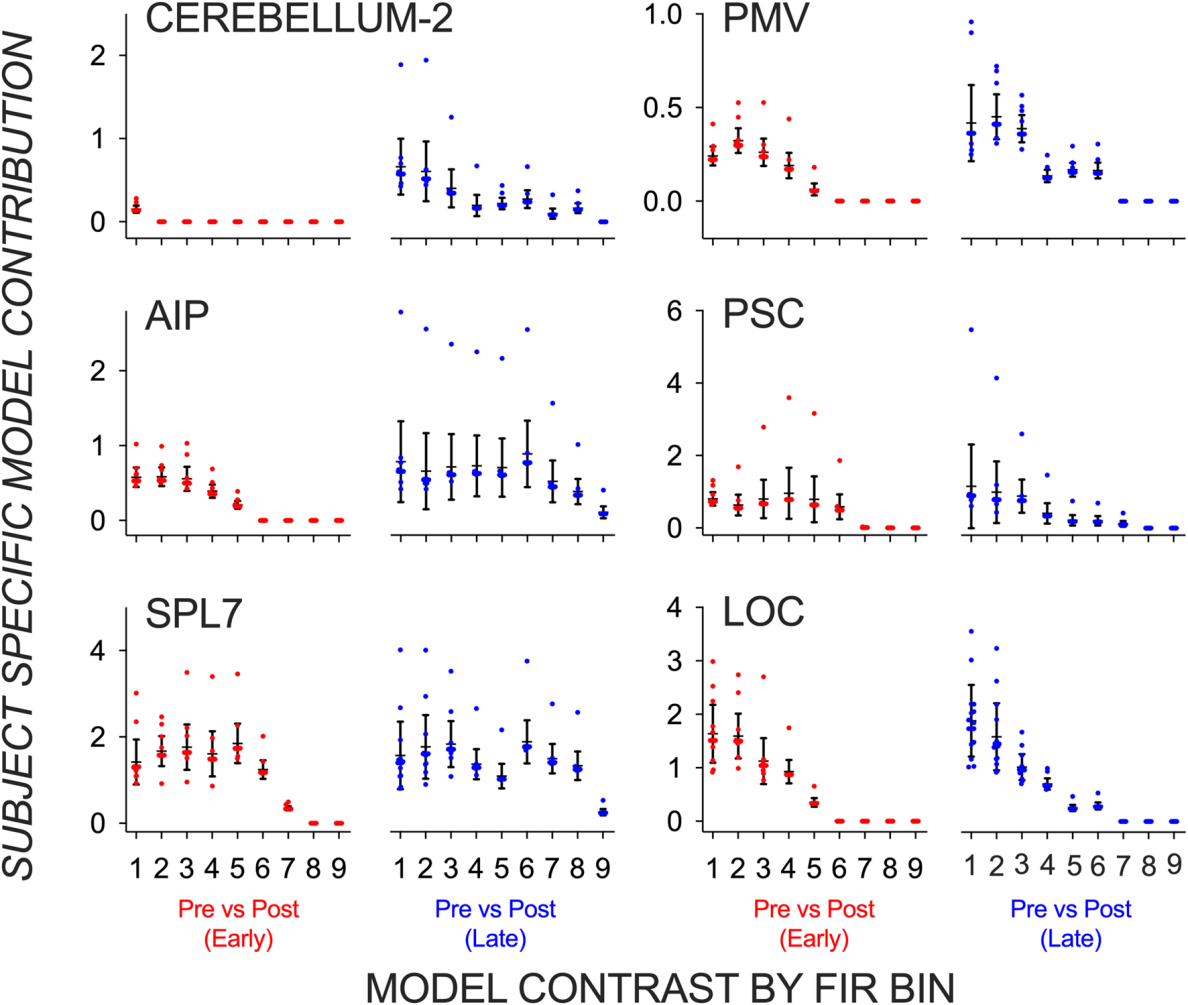
Between-subject consistency to model contributions. Posterior expectation of a hyperparameter from all subjects grouped in a box and whisker plot according to each of the 18 model components or contrasts of interests: pre vs. post-switch trials during early (in red) and late learning (in blue) for finite impulse response (FIR) time bins 1 to 9.

The above findings could be driven by individual subjects acting as outliers. To test for consistency across subjects, we plot in Fig. 4 the posterior density over each subject’s contribution to the data. Each data point represents the posterior expectation of a hyperparameter from a single subject, grouped according to each of the 18 model components or contrasts of interests (pre vs. post contrast during early learning for time bin 1 to 9, and the pre vs. post contrast during late learning for time bin 1 to 9). Most subjects contributed similarly to each of the model components, confirming the between-subject consistency of the reported effects.

## Discussion

In this study we investigated the neural substrates that guide adaptation of anticipatory force control in response to fluctuating dynamics of an object-centric dexterous action. Using Bayesian vRSA of deconvolution-modelled fMRI data, multivoxel spatial patterns in prespecified ROIs were compared as subjects learned appropriate anticipatory force control to generate a torque away from a systematically, explicitly and repeatedly switching left- and right-sided CoM. In line with our hypotheses, CoM-specific pattern distances increased with repeated CoM switching exposures and improved learning in later than earlier blocks in one training session on day 1. These effects were present in all ROIs but most prominently in cerebellum and in early time bins before lift onset, corroborating the putative role for the cerebellum in predictive motor control. On day 2, and consistent with consolidation, these appropriate CoM-specific patterns were already present at the start of the session, with little differences between early and later blocks.

This study reveals a temporal evolution of specialized neural representational patterns that accompany the motor learning process of adapting anticipatory force control to the fluctuating dynamics of an object. Minimizing the roll of an object with an asymmetric CoM that recurrently switched between left and right required adapting anticipatory forces in concert with these switches to generate appropriate torques of opposite directions. This learning of force control that is specialized to each CoM was seen to a greater degree in later than earlier blocks of the first training session on day 1. This learning effect was accompanied by neural representational patterns that also became more specialized to each CoM over the course of the first training session. This suggests these regions contain distinct pattern representations for a given CoM that evolved with learning. Critically, we saw the differentiation of CoM-specific patterns prior to lift onset. In the absence of salient visual cues, the behavioral paradigm we used requires subjects to use a sensorimotor memory of their previous experiences to enact anticipatory force control. Therefore, these pre-lift CoM-specific patterns are likely a neural corollary for the memory that allows anticipatory force control and consequent success in minimizing object tilting^9,10^.

It has recently been shown that specialized preparatory activity patterns can be observed in tasks involving sequentially learned different curl fields^16^. This, as well as our results, supports the idea that during anticipatory planning of force control distinct neural states with separable motor memories are a necessary precursor for achieving success in tasks with opposing perturbations^17^. Learning-related emergence of distinct multivoxel activity patterns have also been documented with motor sequence learning paradigms, where training was performed over far longer learning periods^18,19^. It is remarkable that in our study, CoM-specific patterns emerged more rapidly and were still present 24 h later. To our knowledge, this is the first MVPA study to document a retained neural representational pattern 24 h later alongside improved behavioral effects. We propose that this pattern in fact reflects a learned memory for anticipatory force control that contributes to improved behavioral performance.

Our data offer several additional controls that strengthen the claim that specialization of activity patterns over learning contributes to success. First, we show a lack of evidence for CoM-specialization in neural representational patterns in early blocks when subjects were not yet correctly anticipating forces after a CoM switch. Second, the behavioral improvement on day 2, where subjects performed better in minimizing roll after CoM switches in early blocks, was also accompanied by CoM-specific patterns that more closely resembled that in later blocks. Finally, the fact that there were little to no differences in contrasts of within CoM patterns in early and later blocks rules out that the evolution of CoM-specific patterns is a function of some other time-modulated factor that is unrelated to the learning of CoM-specific force control.

Pre-lift CoM-specific patterns that evolved with learning were most pronounced in cerebellum, which fit with theoretical models of sensorimotor control that emphasize its predictive capacities^20–23^. The basic idea centers on the cerebellum operating as a forward model using efferent copies of motor commands to predict sensory consequences of actions. Error signals are then conveyed back to the cerebellum reflecting a mismatch between the predicted and actual outcome. These signals allow rapid adjustments in the motor output, and are essential for learning to refine future sensory predictions and reduce the prediction error signal on subsequent movements, which we show here can be called upon a day later following training. Our behavioral paradigm relies especially on the use of a forward model for anticipatory control since visual feedback of object properties are not salient. Given the average delay of the hemodynamic response to peak approximately 4 to 6 s later, activity in most of our time bins (up to at least bin 7) would predominantly relate to activity occurring before the object is even lifted. Therefore, the patterns we contrast in vRSA reflect activity that initially contribute to anticipatory behavior reflective of failure (i.e. large roll, in early blocks) and then success (i.e. minimal roll, in late blocks), rather than activity resultant of the error already occurring after feedback of the object properties become available (i.e. the error signal). In other words, these anticipatory representational patterns are temporal precursors to behavioral error (in early) and success following object lifting. In cerebellum, this could be reflective of a predictive process that would lead to an error or success (e.g. an incorrect or correct prediction of the sensory consequence).

The neural representation patterns that result in behavioral error and success offer insights into the longstanding theoretical question of why humans are ineffective at adjusting their behavior to an explicitly known fluctuating environment or object during early sensorimotor learning^7,8,24–26^. Errors, despite explicit awareness, have been attributed to a ‘negative transfer’ effect in behavioral work, whereby subjects persist with, or attempt to use the same anticipatory memory representation that was initially acquired. This was shown as a copying of the motor behavior from the trial preceding the context change on the first trial in the new context, e.g. applying same lift force distribution on the post-switch trial as on the pre-switch trial^24^. Our data do not entirely fit with this hypothesis. While in cerebellum there is a predominant loss of evidence that representational patterns on error trials following a CoM switch were different to the preceding trial, there is still *some* differentiation in early time bins (on both day 1 and day 2), albeit of smaller magnitudes. Similarly, in cortical regions, there was evidence albeit of lower magnitude than later blocks that the error trials during early blocks were different in their representational structure than trials preceding the switch (particularly in early time bins). This suggests that subjects are not simply persisting with or accessing the previously learned representations. This is at least the case during the time points where there *is* evidence of CoM-specific differentiation. Instead, it seems that the temporal dynamics of CoM-specific differentiation are key for learning to successfully adapt force control. Our results show that CoM pattern differentiation leading to error trials is not occurring at the same magnitude and/or not at the right time or duration as that leading to success trials. In cerebellum, the differentiation flattens out after time bin 1, and in later time bins in cortical regions in early than later blocks (see Fig. 3). That the timing of CoM-specific patterns for CoM-specific anticipatory force control matters, especially in cerebellum, is consistent with studies showing deficits in timing of predictive force control in patients with cerebellar degeneration^27^. This supposition fits well those that ascribe the cerebellum as an operator of timing (see^28^ for a review). In addition, it is worth questioning what CoM-specific differences reflect if they are not reflective of success (e.g. the CoM-specific differences, although of smaller magnitude than that seen later, in early blocks when errors are present). It is possible that at least *some* of these early CoM differences are neural correlates of the perceptual awareness of the CoM switch, but without sufficient experience, there is an inability to instantiate the correct representation for successful force control.

Finally, we briefly discuss the slight increases in evidence that within-CoM patterns vary more during early blocks of trials on day 1, the variability of which dissipates over the course of learning. This finding is reminiscent of studies finding regions performing the same behavioral function but with less presynaptic activity (i.e. an effect of ‘neural efficiency’)^18^. Nevertheless, we apply caution to ascribe the within-CoM activity pattern changes between early and later blocks in the absence of a behavioral change to a ‘neural efficiency’ effect. It is not possible to know whether less computational resource is necessary to compute the same function in our data. As has recently been debated, without a formal means of testing neural efficiency, such an explanation for a neural phenomenon seems rather vacuous and simply another descriptor of the data^29^. Furthermore, although the way we measured behavior showed no change in early blocks of the same CoM, the way in which roll minimization is achieved could have varied within different blocks during early learning—e.g. moving from rule-based to automatic, auto-pilot strategies. These changes could explain the slight within-CoM activity pattern differences during early learning. Nevertheless, whatever these within-CoM activity pattern differences are reflective of, they were substantially minimal compared to our main contrasts of interest, and thus not a cause of concern in being a main driver of the noticeably larger early to late between-CoM-specific effects.

In summary, the current study revealed an emergence of CoM-specific neural representations to successfully anticipate force control during object lifting and this pattern was also present 24 h later. The findings reveal a neural substrate that enable humans to predictively and adaptively manipulate objects with dynamically fluctuating properties. These substrates may vary depending on the availability of visual cues^30^ and depending on more static object properties (e.g. weight). Predictive lift force control for object weight might be similarly represented since many of the regions sampled here (e.g. PMv, AIP, PSC, and cerebellum) have been shown to be sensitive to lift force differences based on object weight^31,32^. On the other hand, the feedback control of force direction (increasing vs. decreasing) has been shown to be differentially represented in spatially separate regions^33^.

## Methods

### Participants

16 right-handed healthy adults (median age: 22; range: 19–38; 14 women) with normal or corrected to normal vision participated in this study, and gave informed consent. The study was approved by the Human Subjects Committee, Office of Research, University of California, Santa Barbara. All research was performed in accordance with relevant guidelines and regulations.

### Materials, design, and procedure

#### Materials

The custom-made Plexiglass object (see Fig. 1) was shaped like an inverted T with a vertical column (height: 13.0 cm; width: 3.4 cm; depth: 5.0 cm) with circular grasp surfaces on both sides (diameter: 1.5 cm; between grasp distance: 8 cm), and a horizontal flat base (height: 0.5 cm; width: 18.0 cm; depth: 5.0 cm). A lead block (height: 2.7 cm; width: 5.0 cm; depth: 3 cm; mass: 441 g) was placed on the horizontal base on the left or right side (condition-dependent) of the vertical column creating an asymmetric CoM, which was concealed by black covers (height: 3.4 cm; width: 7.2 cm; depth: 5.0 cm).

The object and a button box were at arm’s length on a wooden table over the hips. The object was rotated 30° in an anti-clockwise direction with respect to the frontal plane, which pilot work showed minimized the wrist’s biomechanical constraints that influence object roll in a supine position (e.g. wrist stiffening). A mirror was attached to the head coil such that subjects had a full and clear view of the object, button box, and their hand at all times.

Vertical height and roll of the object were measured with a two-camera MRI-compatible motion tracking system (Precision Point Tracking System; Worldviz; frame rate: 150 Hz; camera resolution: 640 × 480 VGA; spatial accuracy at focal distance: submillimeter) and with two near-infrared LED markers that were affixed to the vertical column. The total mass of the object was 688 g (torque = 223 Nmm).

#### Experimental design and procedure

The experiment had two within-subject conditions (see Fig. 1): manipulating the object with a left and a right CoM. After standardized instructions and lifting a water bottle according to the task’s audio cues, subjects lifted the object with the main goal of minimizing the object roll at all times. The experimenter rotated the object every four trials such that subjects were exposed to 4 blocks of trials containing 4 trials manipulating a left and a right CoM, respectively. Block order was counterbalanced across subjects. Apart from the very first trial, subjects were informed about the CoM of the upcoming block of trials. On each trial, subjects held down the button with the palm of their right hand in a relaxed position until an audio start cue instructed them to reach, grasp and lift the object, hold it at the height of a marker (4 cm) until an audio stop cue (4 s after the start tone), after which the object was to be placed in its original position and the hand returned to the button. An error cue was subsequently played if the object rolled more than 5° in either direction during the trial. The start cue of the first trial coincided with the beginning of a new functional image. This procedure was repeated 24 h later in 10 subjects.

Anatomical and functional MRI data were acquired using a Siemens 3 T Magnetom Prisma Fit (64-channel phased-array head coil). High-resolution 0.94 mm isotropic T1-MPRAGE (TR = 2,500 ms, TE = 2.2 ms, FA = 7°, FOV = 241 mm) sagittal sequence images were first acquired of the whole brain. Following this, subjects performed the object manipulation task while BOLD contrast was measured with a CMRR multiband (University of Minnesota) T2*-weighted echo planar gradient-echo imaging sequence (TR = 400 ms, TE = 35 ms, FA = 52°, FOV = 192 mm, multiband factor 8). Each functional image comprised 48 slices which were acquired parallel to the AC-PC plane (3 mm thick; 3 × 3 mm in-plane resolution). The behavioral experimental protocol was interleaved between 25 TRs at the start and at the end of the functional run.

### Statistical analyses

#### Kinematic data processing and analyses

Data collected were filtered using a 4th order Butterworth filter with a cutoff frequency of 5 Hz. Object lift onset was defined as the timepoint at which the vertical position of the object went above 1 mm and remained above this value for 20 samples. Object roll was defined as the angle of the object in the oblique plane. Peak object roll was recorded shortly after lift onset (~ 250 ms) before somatosensory feedback resulted in corrected feedback responses to counter object roll. Trials with object roll > 5° were classified as errors. We ran a two-way ANOVA to examine the effect of a CoM switch (2 levels, pre- vs. post-CoM switch trial) and block (7 levels; block 1 to 7) on object roll and corrected for multiple comparisons using the Holm-Šídák test for day 1 and day 2 data. The Greenhouse Geisser correction was applied for sphericity violations.

#### MRI data processing and analyses

MRI data were pre-processed and analyzed using SPM12 (Wellcome Trust Center for Neuroimaging, London, UK) and FSL (https://fsl.fmrib.ox.ac.uk/fsl/fslwiki/)^34^. Using SPM, subjects’ functional images were spatially realigned to a mean image using 2nd degree B-spline interpolation, and coregistered to the T1. We used the SUIT SPM toolbox for between-subject normalization of the cerebellum^35–38^ (interpolation: trilinear, voxel size: 2 × 2 × 2 mm) and SPM’s normalize function for between-subject normalization of the rest of the brain.

A Bayesian implementation of RSA, vRSA^13^ was applied using an adaptation of the Matlab script DEMO_CVA_RSA.m available in SPM12. As specified in the technical note on vRSA, this method can be used to assess the evidence for condition-specific patterns of responses distributed over voxels in a set of predefined regions. It is possible to use classification accuracy derived from a decoding approach to assess condition-specific information in activity patterns. However, its conversion of a continuous measure of dissimilarity into a binary decision is a less informative and less reliable measure of brain representations than the underlying continuous measures offered by encoding approaches such as the one used here^39,40^.

To do vRSA, a deconvolution-based GLM was computed, selecting a finite impulse response (FIR) with the onsets for each of the experimental conditions set to 800 ms before lift onset (window length: 7.2 s; order: 800 ms). This time window and order was selected to sufficiently track activations before lift onset and through the peak of the HRF relating to lift onset, which was assumed to occur 4–6 s after lift onset. The RobustWLS Toolbox in SPM^41^ was used down-weight functional images with high noise variance to account for movement artifact. The GLM was set up with 4 conditions for each block with both CoMs: pre-switch trial 1, pre-switch trials 2–4, post-switch trial 1, post-switch trials 2–4. Error trials in pre- or post-switch trials after the first trials were assigned to an extra error condition trial, which were modelled but not used in further analyses.

GLM-derived beta values for each of the conditions were extracted from regions of interest (ROIs) using FSL’s fslmeants. We selected pre-defined cerebellar and cortical ROIs that have previously been shown to be sensitive to differences when manipulating objects of different torques^10^: superior parietal lobule 7 (SPL7), anterior intraparietal area (AIP), primary somatosensory areas (PSC/SI), and lateral occipital cortex (LOC) were extracted from the SPM Anatomy Toolbox^42–44^. Ventral premotor area (PMv) were free-drawn on a standardized surface mesh in SUMA^45^ based on predefined anatomical parcellations^46–49^, which were projected to standard MNI space and mapped backed to the subject’s T1-weighted image^50^. Cerebellar ROI 2 was extracted from a recently published cerebellar functional atlas^51^. See Fig. 3 for ROIs mapped onto a cortical surface.

Similar to a more traditional RSA approach, vRSA starts by comparing between-condition distances in spatial voxel patterns in a given ROI. Whereas a traditional RSA would express such differences in a representational dissimilarity matrix, in vRSA, they are expressed in terms of second-order similarity or covariance matrices. These matrices describe the relation between activity patterns in terms of each of the stimuli or conditions of interest. As previously specified, the method evaluates the contribution of multiple contrasts to a region’s activity pattern, while taking into account all specified contrasts and their interactions. Thus, results could show a region’s sensitivity to more than one contrast. The outcome of each contrast is a log evidence value, i.e. Bayes factor. This value quantifies the evidence for a given contrast to contribute to dissociable activity patterns in a given ROI. The Bayes factor is central to testing hypotheses using Bayesian statistics, which offers a continuous measure of evidence for H0 (i.e. there is no difference in contrasting patterns) and H1 (i.e. there is a difference in contrasting patterns)^52^. Bayes factor values above 1 favor H1 over H0, values below 1 favor H0 over H1, and a value of 1 favors neither H1 nor H0. In addition, similar to that provided for effect sizes, rough guidelines have been put forth to interpret the magnitude of Bayes factor. A Bayes factor of approximately 3 has been suggested to match a “substantial” amount of evidence that a contrast of interest contributes to a region’s observed response pattern^14,15^.

In the present study, we used vRSA model comparisons to assess the evidence for a region to be sensitive to differences between pre- and post-switch trials in earlier as well as that during later blocks in each of the 9 FIR time bins. We have replicated twice that these regions are sensitive to CoM differences on successful trials before the object is lifted and feedback of object properties become available. In this paper, we assessed what happens to this sensitivity when one trial is no longer a success trial. As shown, behaviorally the post-switch trial after three CoM switches were statistically different (i.e. errors) than that which came before it, whereas the last four switches were not. Thus, we tested two model comparisons comparing an ROI’s sensitivity to differences between pre- and post-switch trials when the latter is an error (blocks 1 to 3) and a success (blocks 4 to 7), respectively, in each FIR bin. As a control measure, two additional model comparisons assessed an ROI’s sensitivity to differences between early and later blocks of the same CoM (e.g. block 1 vs. 3, 2 vs. 4 vs. blocks 5 vs. 7, 6 vs. 8). Based on previous and current behavioral results reflecting ultrafast adaptation of very rapidly minimizing roll after the first exposure of a new CoM, we expected minimal differences in patterns of the same CoM during early and late blocks.

All analyses were repeated 24 h later on a subset of subjects. Note that these subjects performed a similar task with the object in minimizing the same torque after this first session before returning for day 2 testing: 6 functional runs of 28 trials each (those results pertain to an alternative question and was published recently^10^). As before, a conservative uninformed approach was adopted, selecting default hyperprior values from the spm_reml_sc.m script (− 32, 256) because we had little evidence or prior beliefs as to how error might be represented. Further details of the vRSA implementation, its rationale over more traditional univariate and multivariate fMRI analyses, and why multiple comparisons are not needed can be found in^10^.

## Supplementary information

Supplementary file 1

## Data Availability

The source data can be found in the supplementary materials. Raw and processed MRI images and the modelled beta images for all ROIs are available from the corresponding author on reasonable request.
